# Supplementary material for: Analytical ab initio hessian from a deep learning potential for transition state optimization
Source: Nat Commun. 2024 Oct 14;15:8865. doi: 10.1038/s41467-024-52481-5 (PMC11473838; doi:10.1038/s41467-024-52481-5)
Supplement: Supplementary file 1 — Supplementary Information [file 41467_2024_52481_MOESM1_ESM.pdf]

Supplementary Information

S1 Supplementary Figures

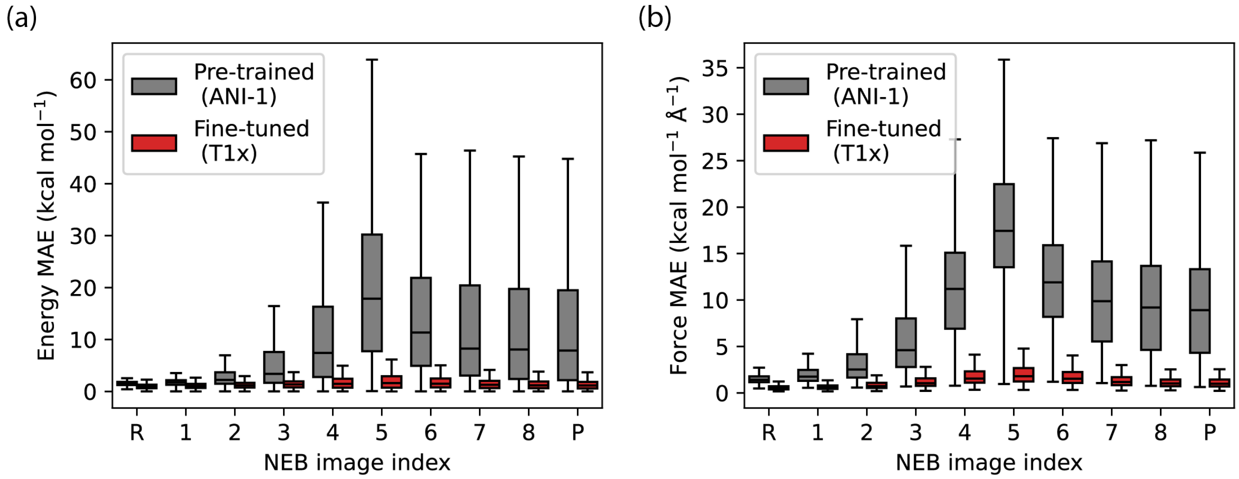

Figure S1: *Statistical performance of pre-trained and fine-tuned models on energies and forces.* The distribution of the prediction error in (a) potential energies and (b) forces along the reaction coordinate of unseen molecules in unseen reactions in the T1x test set. The pre-trained model well predicts near-equilibrium molecular properties but fails significantly around the transition state (TS). The fine-tuned model after TS-rich training captures the potential much more accurately across the reaction barriers. Outliers beyond 1.5 interquartile range are not shown. NEB: nudged elastic band; MAE: mean absolute error.

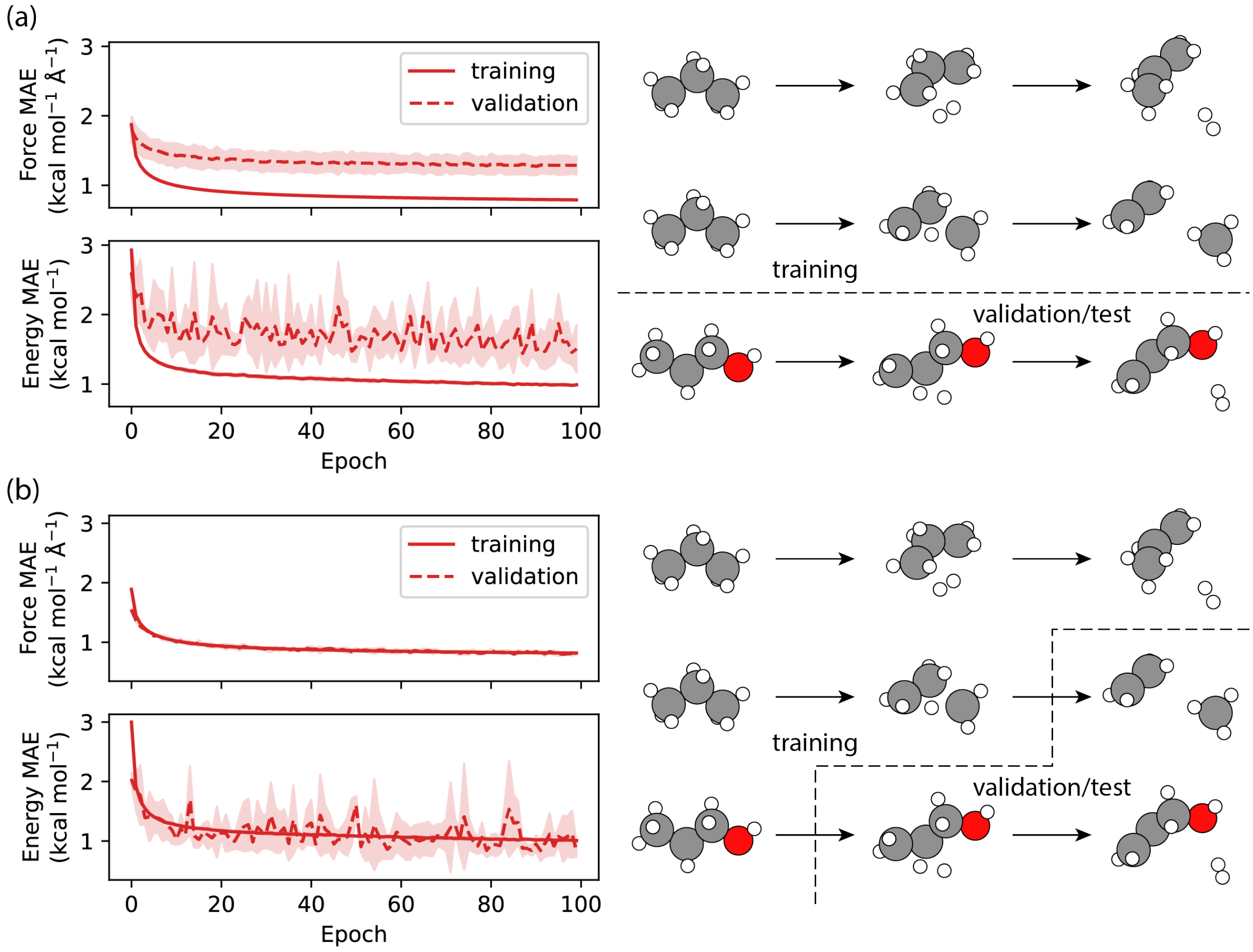

Figure S2: *Model training curve on T1x dataset.* (a) The models trained on compositional splits (minimal data leakage) effectively represent the behaviors on unseen molecules and unseen reactions. (b) The models trained on conformational splits (maximal data utility) have seen all reactions in the T1x-aug dataset and possess the highest level of chemical knowledge possible. MAE: mean absolute error.

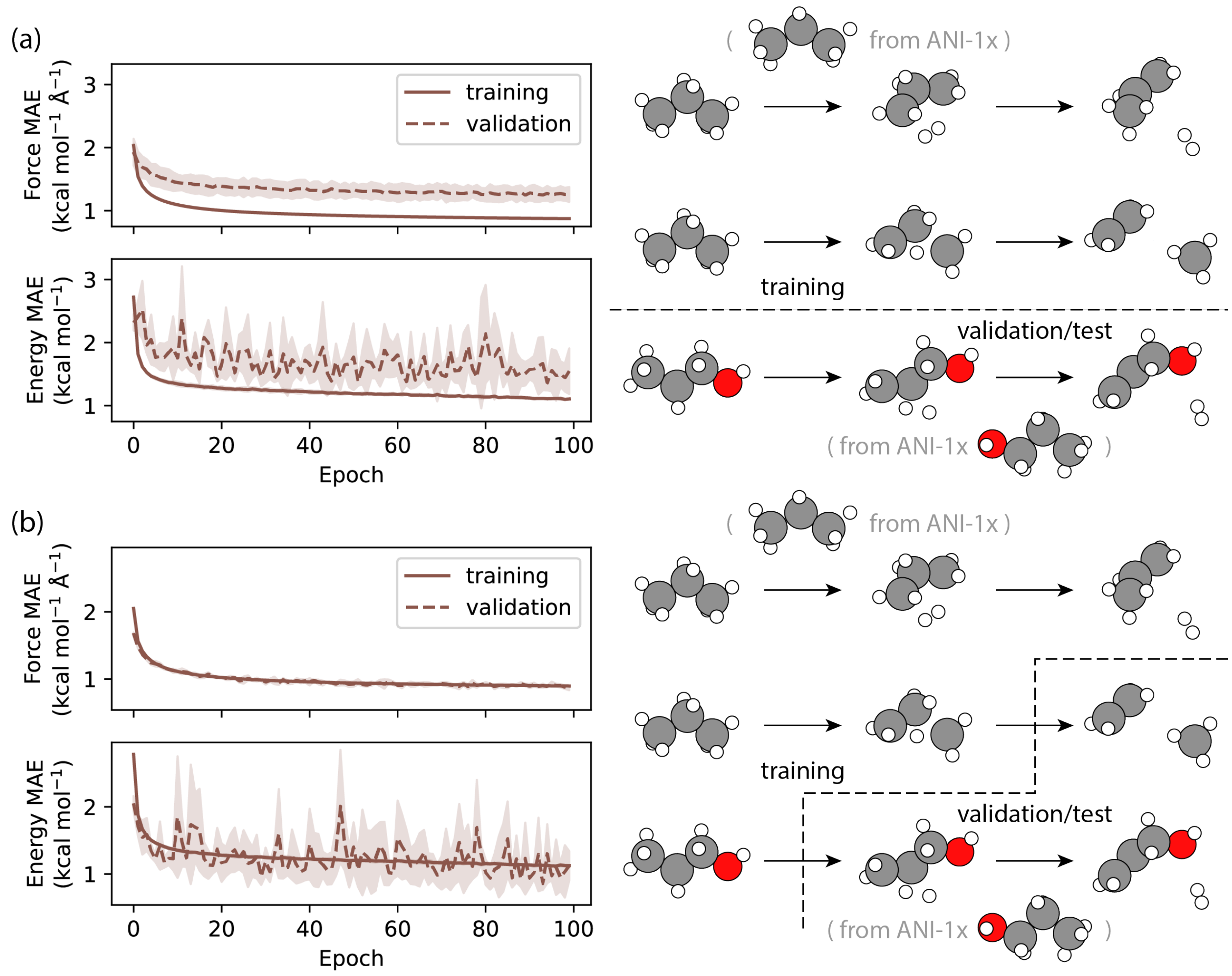

Figure S3: *Model training curve on T1x-aug dataset.* (a) The models trained on compositional splits (minimal data leakage) effectively represent the behaviors on unseen molecules and unseen reactions. (b) The models trained on conformational splits (maximal data utility) have seen all reactions in the T1x-aug dataset and possess the highest level of chemical knowledge possible. MAE: mean absolute error.

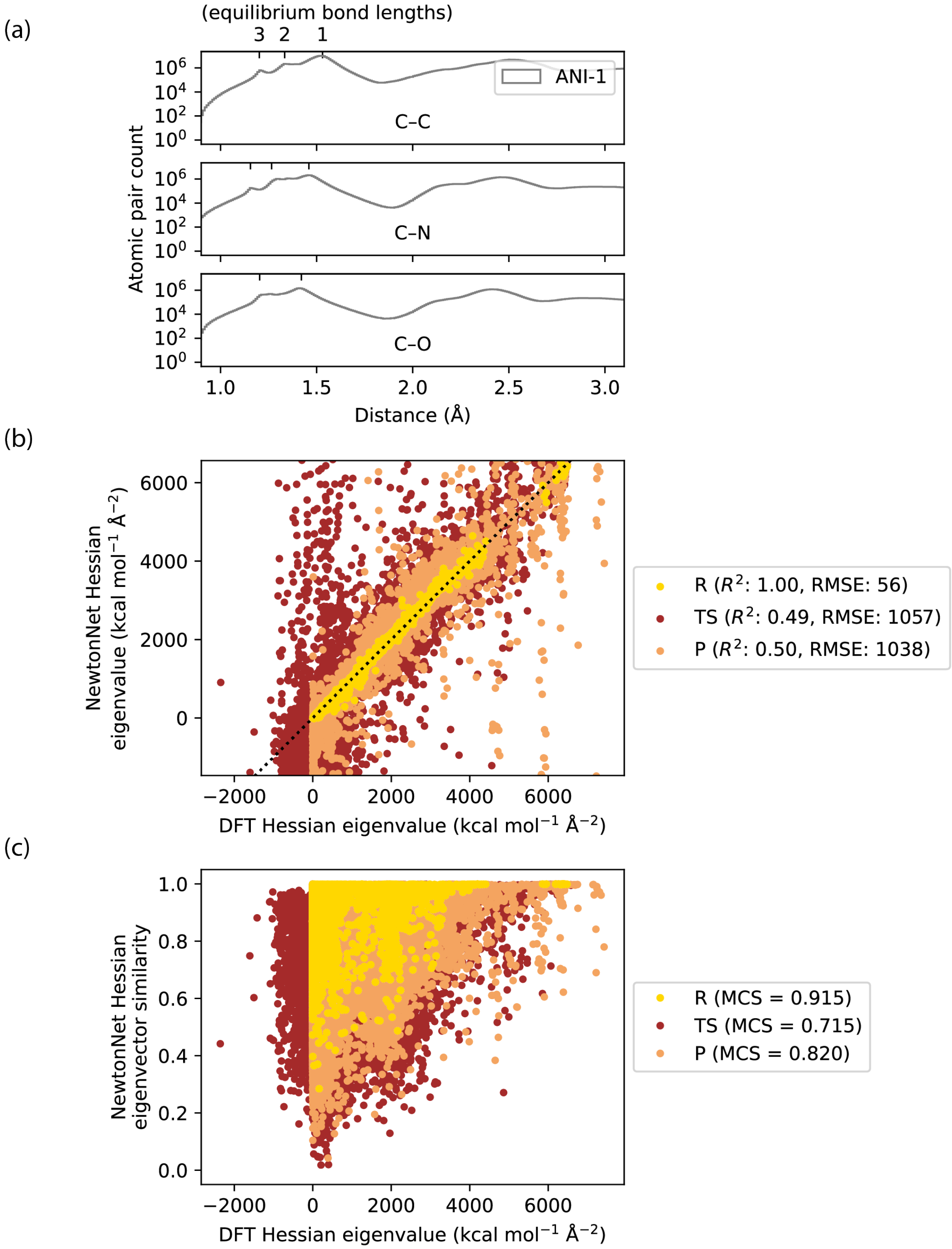

Figure S4: *Hessian predictions of the pre-trained model using the ANI-1 data set.* (a) The distribution for C-C, C-N, and C-O distances, as well as marks of single, double, and triple bond distances of bonds in the ANI-1 dataset. (b) The Hessian eigenvalues predicted by the pre-trained Newton model compared to DFT. (c) The Hessian eigenvectors predicted by the pre-trained Newton model compared to DFT. DFT: density funcitonal theory; RMSE: root-mean-square error; MCS: mean cosine similiarity.

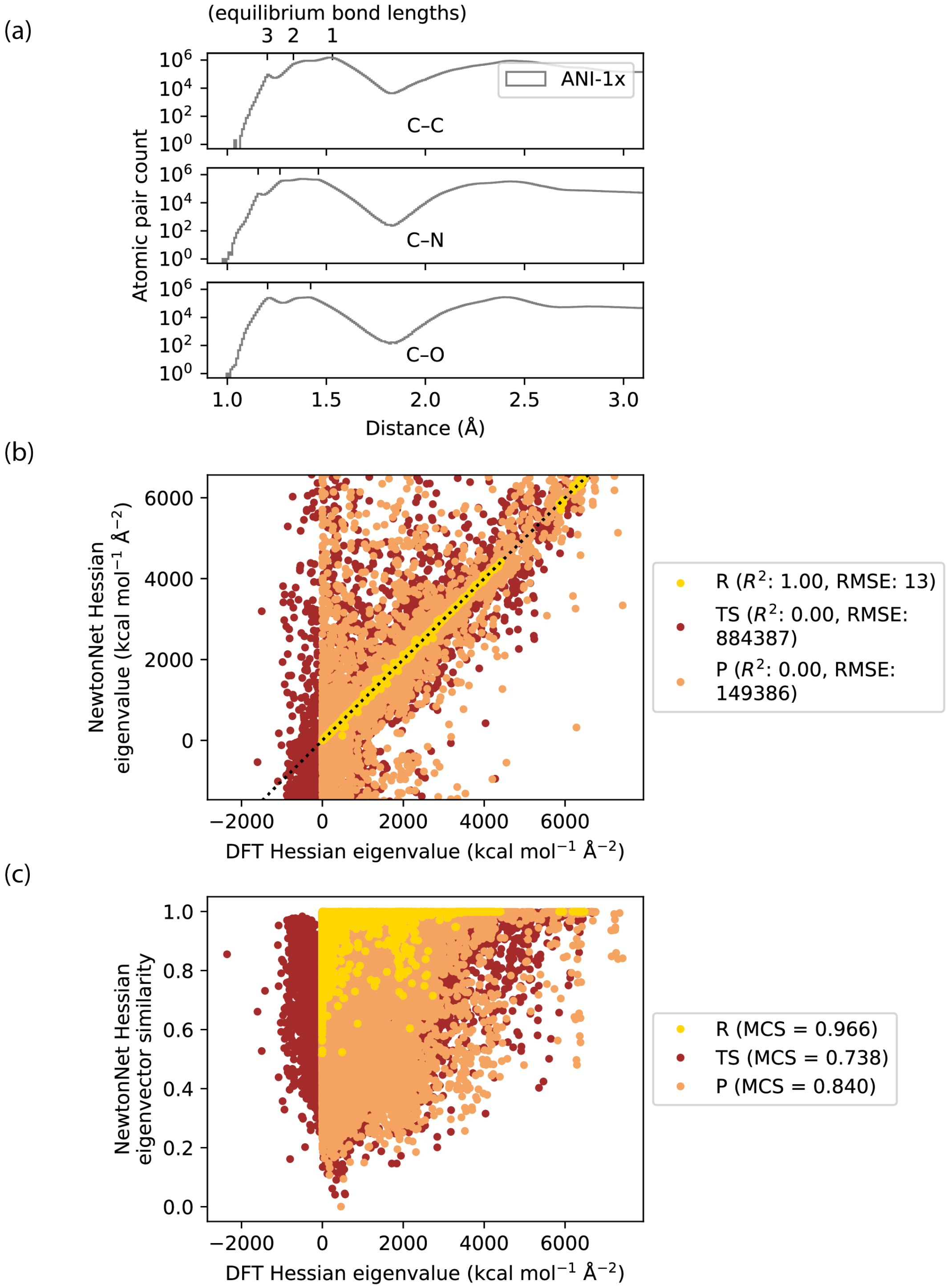

Figure S5: *Hessian predictions of the pre-trained model using the ANI-1x data set.* (a) The distribution for C-C, C-N, and C-O distances, as well as marks of single, double, and triple bond distances, in the ANI-1x dataset. (b) The Hessian eigenvalues predicted by the pre-trained Newton model compared to DFT. (c) The Hessian eigenvectors predicted by the by the pre-trained Newton model compared to DFT. DFT: density funcitonal theory; RMSE: root-mean-square error; MCS: mean cosine similiarity.

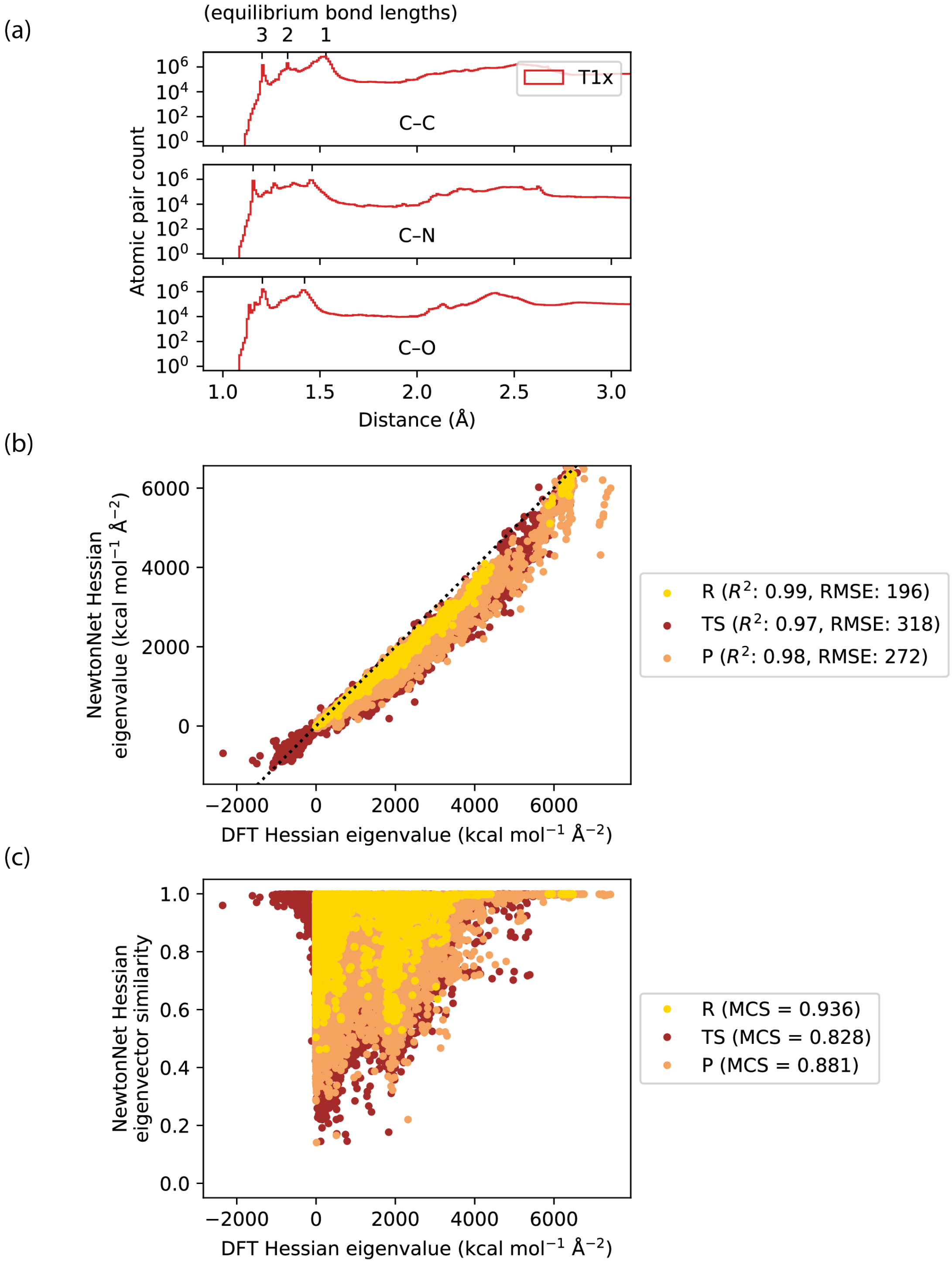

Figure S6: *Hessian predictions of the fine-tuned model using the T1x data set.* (a) The distribution for C-C, C-N, and C-O distances, as well as marks of single, double, and triple bond distances, of the T1x dataset. (b) The Hessian eigenvalues predicted by the fine-tuned Newton model compared to DFT. (c) The Hessian eigenvectors predicted by the by the fine-tuned Newton model compared to DFT. DFT: density funcitonal theory; RMSE: root-mean-square error; MCS: mean cosine similarity.

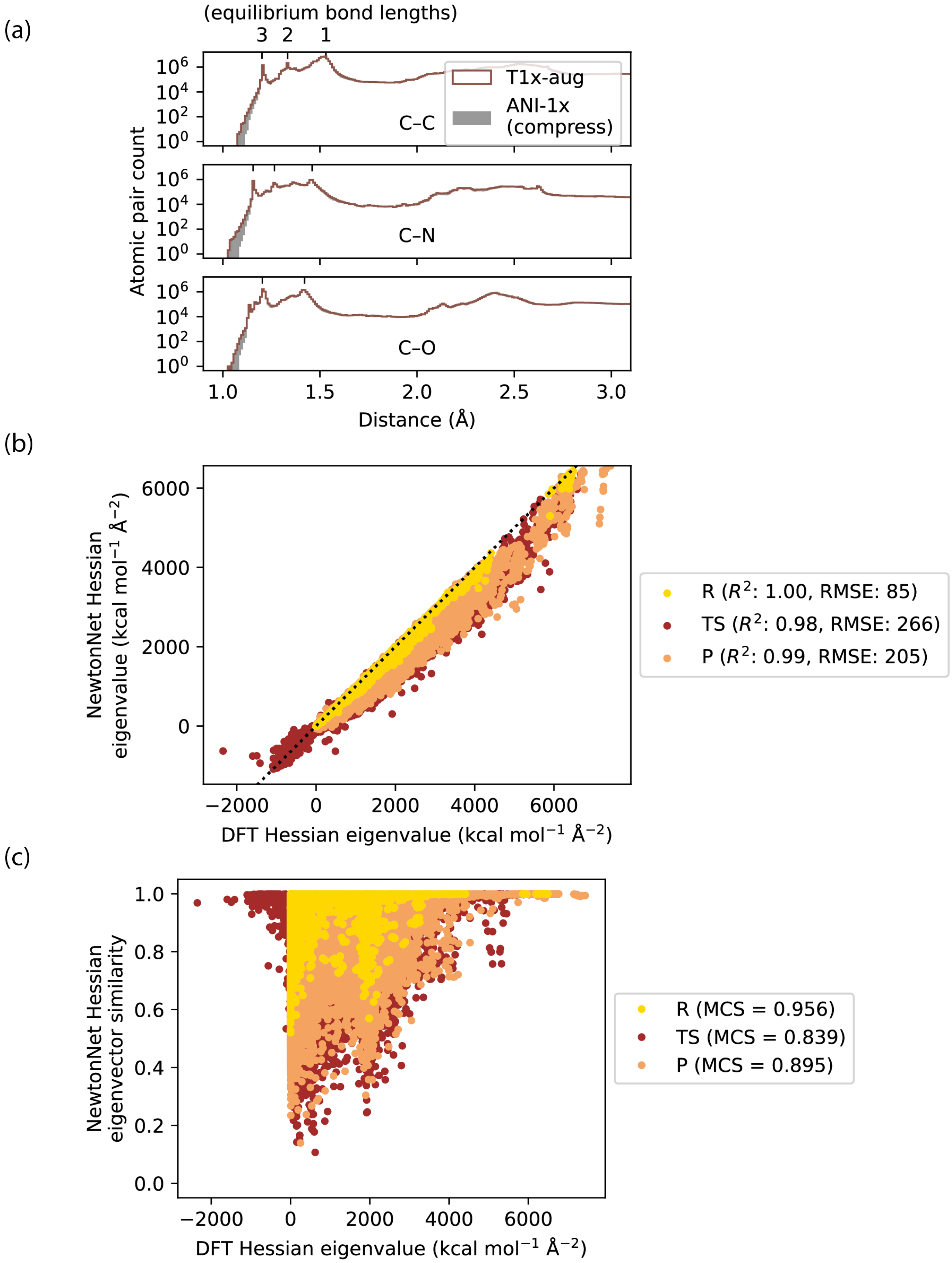

Figure S7: *Hessian predictions of the fine-tuned model on the aug-T1x data set with 1 million extra data points.* (a) The distribution for C-C, C-N, and C-O distances, as well as marks of single, double, and triple bond distances, of the compression-augmented T1x dataset (1 million extra data points). (b) The Hessian eigenvalues predicted by the fine-tuned Newton model compared to DFT. (c) The Hessian eigenvectors predicted by the by the fine-tuned Newton model compared to DFT. DFT: density funcitonal theory; RMSE: root-mean-square error; MCS: mean cosine similarity.

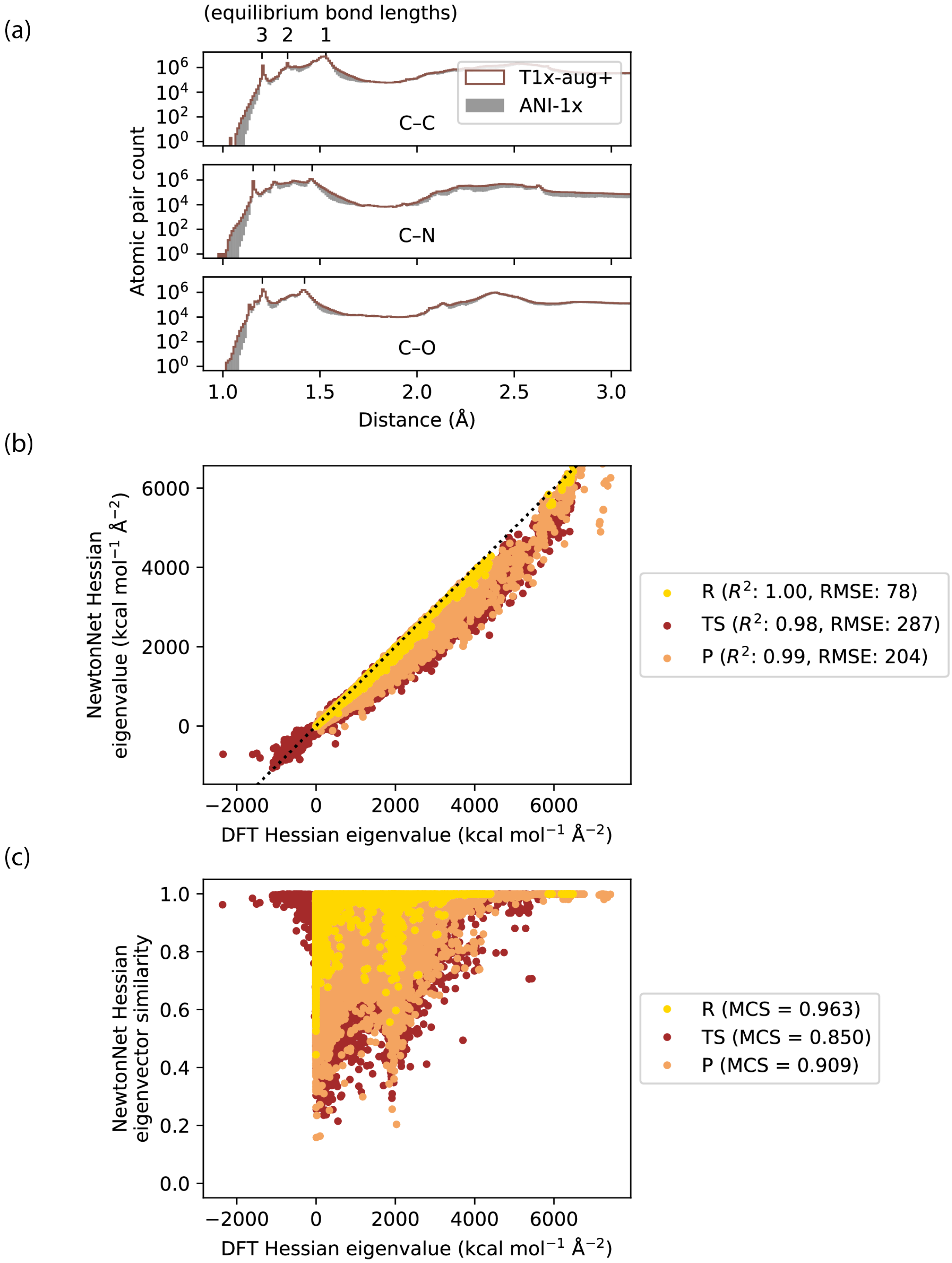

Figure S8: *Hessian predictions of the fine-tuned model with 4 million extra data points.* (a) The distribution for C-C, C-N, and C-O distances, as well as marks of single, double, and triple bond distances, of the equilibrium-augmented T1x dataset (4 million extra data points). (b) The Hessian eigenvalues predicted by the fine-tuned Newton model compared to DFT. (c) The Hessian eigenvectors predicted by the by the fine-tuned Newton model compared to DFT. DFT: density funcitonal theory; RMSE: root-mean-square error; MCS: mean cosine similarity.

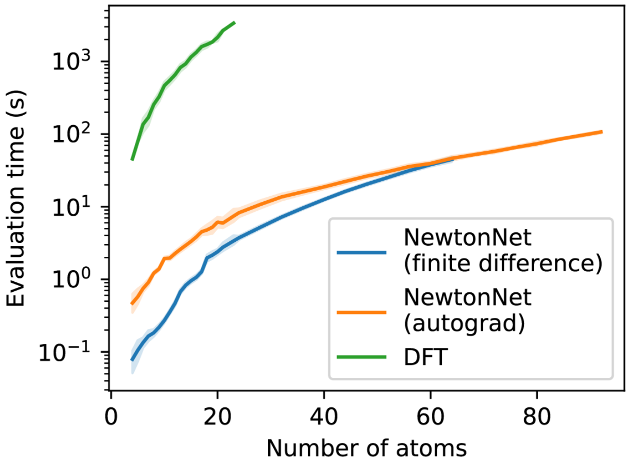

Figure S9: *Computational efficiency improvement of ML Hessian calculations using finite difference and analytical formulations vs. DFT.* The ML Hessian can be calculated ∼3 orders of magnitude faster compared to the same DFT level of theory used for training, either using finite difference method or automatic differentiation. Interestingly, the finite difference method demonstrates a slight efficiency advantage over automatic differentiation in small systems, but as the system grows large, the automatic differentiation becomes more time- and memory-efficient, as it requires generating only one first-order graph instead of multiple graphs for each dimension. DFT: density funcitonal theory; ML: machine learning.

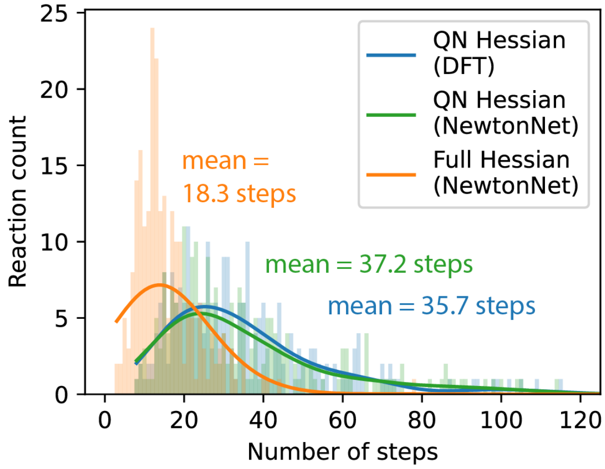

Figure S10: *Efficiency improvement of full-Hessian TS optimization.* Using the full Hessians from ML potential improves the TS optimization efficiency over the Sella benchmark reactions. DFT: density funcitonal theory; ML: machine learning; QN: quasi-Newton. Lines are kernel density estimate fits to the histogram data.
